# Biodegradable nanocarriers coated with polymyxin B: Evaluation of leishmanicidal and antibacterial potential

**DOI:** 10.1371/journal.pntd.0007388

**Published:** 2019-05-01

**Authors:** Juliana Souza Ribeiro Costa, Marília Medeiros, Edite Harumi Yamashiro-Kanashiro, Mussya Cisotto Rocha, Paulo Cesar Cotrim, Marco Antonio Stephano, Marcelo Lancellotti, Guilherme Diniz Tavares, Laura Oliveira-Nascimento

**Affiliations:** 1 Department of Biochemistry and Tissue Biology, Biology Institute, University of Campinas, Campinas, São Paulo, Brazil; 2 Faculty of Pharmaceutical Sciences, University of Campinas, Campinas, São Paulo, Brazil; 3 Tropical Medicine Institute, University of São Paulo, São Paulo, São Paulo, Brazil; 4 School of Pharmaceutical Sciences, University of São Paulo, São Paulo, São Paulo, Brazil; 5 University of Campinas, Campinas, São Paulo, Brazil; Azrieli College of Engineering Jerusalem, ISRAEL

## Abstract

Most treatments of leishmaniasis require hospitalization and present side effects or parasite resistance; innovations in drug formulation/reposition can overcome these barriers and must be pursued to increase therapeutic alternatives. Therefore, we tested polymyxin B (polB) potential to kill *Leishmania amazonensis*, adsorbed or not in PBCA nanoparticles (PBCAnp), which could augment polB internalization in infected macrophages. PBCAnps were fabricated by anionic polymerization and analyzed by Dynamic Light Scattering (size, ζ potential), Nanoparticle Tracking Analysis (size/concentration), vertical diffusion cell (release rate), drug incorporation (indirect method, protein determination) and *in vitro* cell viability. Nanoparticles coated with polB (PBCAnp-polB) presented an adequate size of 261.5 ± 25.9 nm, low PDI and ζ of 1.79 ± 0.17 mV (stable for 45 days, at least). The 50% drug release from PBCAnp-polB was 6–7 times slower than the free polB, which favors a prolonged and desired release profile. Concerning *in vitro* evaluations, polB alone reduced *in vitro* amastigote infection of macrophages (10 μg/mL) without complete parasite elimination, even at higher concentrations. This behavior limits its future application to adjuvant leishmanicidal therapy or antimicrobial coating of carriers. The nanocarrier PBCAnp also presented leishmanicidal effect and surpassed polB activity; however, no antimicrobial activity was detected. PolB maintained its activity against *E*. *coli*, *Pseudomonas* and *Klebsiella*, adding antimicrobial properties to the nanoparticles. Thus, this coated drug delivery system, described for the first time, demonstrated antileishmanial and antimicrobial properties. The bactericidal feature helps with concomitant prevention/treatment of secondary infections that worst ulcers induced by cutaneous *L*. *amazonensis*, ultimately ending in disfiguring or disabling lesions.

## Author summary

Cutaneous leishmaniasis form disfiguring lesions that leads to depression and social isolation. Both consequences result often in economic and education loss for communities already short on resources. The actual treatment aims to eliminate the protozoa disease-vector. Yet, it presents undesired effects or parasite resistance, besides the need for intravenous infusions and hospitalization. There is a few topical leishmanicidals but none with prolonged action. As highlighted, no ideal therapy exists; we must keep searching for alternatives, which includes new drugs, vehicles or therapies. For drugs, the most cost-effective way comprises testing approved molecules for other diseases, such as the antimicrobial polymyxin B (polB). Repositioning a drug has the added benefit of known safety profile and drawbacks, shortening and narrowing the research path. In turn, carriers can decrease side-effects and multiple dosing. They shield and/or improve drug targeting, which in this case means delivering inside macrophages infected with *Leishmania*. An example is poly (n-butyl cyanoacrylate) nanoparticles (PBCAnp), shown as an efficient carrier for targeting an extended drug release. Therefore, this work aimed to evaluate leishmanicidal and adjuvant properties of polB, alone and absorbed onto PBCAnp.

## Introduction

Leishmaniasis is a public health problem in tropical countries, classified as a neglected disease by World Health Organization (WHO) [1]. Phlebotomine sandflies transmit over 20 species of protozoa from the genus *Leishmania*, an intracellular parasite which hosts mammalian macrophages and establishes disease [2]. WHO divides leishmaniasis into three types of infections: visceral, mucocutaneous and cutaneous leishmaniasis (CL) [3].

Nowadays, 70–75% of CL cases are present in 10 tropical countries of the world [4]. CL causes ulcerated lesions in the skin, which can evolve to disfiguration, mutilation and allow secondary infection by bacteria [5]. CL treatment relies on meglumine antimoniate, sodium stibogluconate, pentamidine, amphotericin B deoxycholate and liposomal amphotericin B [6]. However, these drugs present relevant side effects, require hospitalization for intravenous administration and present some reports on drug resistance [7]. Fluconazole, ketoconazole, and miltefosine can also be administrated as an oral treatment. Newer drugs, such as paromomycin are an alternative to topical treatment [6]. With higher disease incidence in low-income regions, industrial investments in drug innovation are less attractive, such as alternative dosage forms [1].

The use of nanoparticles (NPs) as carriers can overcome drug resistance, side effects and unspecific biodistribution of microbicidal agents, which reduce high drug dosage, loss of activity and the need for chemical modifications [8]. Macrophage increased uptake and residence time can also be accomplished with NPs, a desired feature for a carrier to deliver leishmanicidal drugs. Poly butyl-cyanoacrylate nanoparticles (PBCAnp), for instance, are based on a low toxicity synthetic polymer with adequate biodegradation. Moxifloxacin loaded in PBCAnp triplicated drug accumulation inside macrophages, besides the high increase in residence time [9]; gentamicin uptake was quintuplicated with the PBCAnp given to peritoneal macrophages [10]. PBCAnp also holds another important feature: molecules can be adsorbed on its surface by covalent attachment or internalized during polymerization. This flexibility allows surface functionalization or multiple drug loading with different kinetic release profiles [11].

Even with technological approaches (such as NPs) to enhance the leishmanicidal effect, the treatment for CL does not rely solely on parasite elimination. Most secondary infections in CL ulcers present Gram-negative bacteria, as *Klebsiella pneumoniae* and *Pseudomonas aerugionosa* [5], which do not impair leishmaniasis healing; however, *Pseudomonas* secondary infection can be a co-factor in mutilation induced by CL [12], besides inhibition of phagocytosis due to alginate supported biofilms [13]. Treatment for dermal infection caused by *Pseudomonas* and other Gram-negative bacteria includes the antimicrobial peptides called polymyxins, which are also active against few strains of Gram-positive bacteria [14]. Among polymyxins, polymyxin B (polB) is used against *Pseudomonas* infections and briefly reported as an inhibitor of *Leishmania* growth [15]; other cationic antimicrobial peptides were classified as leishmanicidal, but none of them are available in the market [16]. Since peptide activity can decrease due to cell culture medium additives, we fabricated PBCAnp to adsorb the antimicrobial and deliver it inside macrophages. Considering all previous statements, we produced PBCAnp adsorbed with polB to evaluate its leishmanicidal effect and its viability as a functionalized antibacterial carrier.

## Methods

### Materials

N-butyl-cyanoacrylate monomer (10 mg/mL, BBraun, Germany). Dextran sulphate (70,000 Da, Sigma-Aldrich, Denmark). Albumin, bicinchoninic kit (QuantiPro BCA Assay Kit), Dimethyl sulfoxide, ethanol, hydrochloric acid, isopropanol, methanol, MTT (3-(4,5-dimethylthiazol-2-yl)-2,5-diphenyltetrazolium bromide) and sodium hydroxide were from Sigma-Aldrich, MO, USA. Cellulose membrane (Spectra/por 2, 12 to 14 kDa, Spectrapore, USA). M199 medium, RPMI 1640 medium and Müller-Hinton broth were from Vitrocell, Brazil. Rapid panoptic dye (Laborclin, Brazil). Amphotericin B was donated by Cristália, Brazil. Polymyxin B sulphate (500,0000 UI) was donated by Química Haller, Brazil. Distilled water was used for chemical assays and ultrapure water (Milli Q, 18.2 MΩ.cm at 25°C and a TOC value below 5 ppb) for biological experiments and formulations.

### Nanostructure assembling

The PBCAnp synthesis was performed by the emulsion polymerization method [17,18]. 100 μL of n-butyl-cyanoacrylate monomer was added dropwise, under vigorous stirring (800 rpm), to a 10 mL aqueous solution of HCl 0.1 M (pH 2.5) containing 100 mg of dextran 70,000 Da. After 4 hours of stirring, the pH was adjusted to 7.0 ± 0.3 with NaOH 0.1 M. The nanosuspension was filtered (1 μm filter membrane) and centrifuged (14,000 x g, 30 minutes, 25°C) to remove polymer that did not retained a particle structure; the pellet was then re-suspended in ultra purified water for the control carrier formulation (PBCAnp) or in 5 mg/mL polB sulphate solution (PBCAnp-polB), resulting in formulation of nanoparticle suspensions.

### Nanostructure characterization

#### Determination of hydrodynamic diameter of nanoparticles (size) and electrophoretic mobility (Zeta potential)

The average particle diameter was analyzed by Dynamic Light Scattering (DLS) (Zetasizer Nano ZS, Malvern Instruments Ltd, Malvern, England), at 90° angle and 25°C, with samples diluted in ultrapure water (refraction index 1.333 –viscosity 0.8905 cP) for adequate correlation coefficient (0.7 to 1). Zeta potential was analyzed by electrophoretic mobility, also with Zetasizer. Nanoparticle Tracking Analysis (NTA) (Nanosight, Malvern Instruments Ltd, Malvern, England) was performed to determine particle concentration and particle size diameter. Analyses were performed at 25°C, with diluted samples in ultrapure water up to 30–100 particle per frame—10^7^−10^9^ particles/mL. Both analyses were done in triplicate for every time point.

#### Incorporation efficiency (IE)

The IE, also known as entrapment efficiency, was determined by the indirect method, whereas the total amount of drug = 100%. NPs suspensions were separated from the diluent by centrifugation (14,000 x g, 30 minutes, 25°C). The supernatant was then quantified by the bicinchoninic kit, based on colorimetric reaction detected by UV-vis spectroscopy (560 nm). The calibration curve was made with albumin (1.3–20 μg/mL; R^2^ = 0.99), and compared with the same concentrations of polB (R^2^ = 0.99). There was a significant difference between curve inclinations, so the polB curve was used as a reference (S1 Fig). The IE was calculated by the Eq (1). Drug loading (DL) was calculated by the Eq (2). To obtain the total nanoparticle weight, samples were freeze dried.

IE(%)=(Druginthesupernatant/Totaldrug)x100 (1)

DL(%)=(Amountoftotalincorporateddrug/totalnanoparticleweight)x100 (2)

#### Drug release profile

NP drug release profile was analyzed on vertical diffusion cell system, with cellulose membrane (12 a 14 kDa) separating the donor compartment (with 0.4 mL of sample) from the acceptor compartment, buffer pH 7.4 and light stirring (37°C). The samples were withdrawn from the acceptor compartment at predetermined intervals and subjected to drug quantification by the bicinchoninic kit, as described in the incorporation efficiency item. The withdrawn sample volume was immediately replaced by the buffer. The percent release was calculated at each sample withdrawal time, in sextuplicate, with the points subsequently plotted on time x release rate.

#### Morphology by scanning electron microscopy (SEM)

NP morphology was analyzed by SEM, on the equipment JEOL JSM-6340-F. Samples were dehydrated on a silica wafer at room temperature, coated with gold-palladium and analyzed.

### *In vitro* evaluations

#### Leishmanicidal activity

Cell viability of *L*. *amazonensis* promastigotes in the presence of the formulations (24h) was determined by MTT (3-(4,5-dimethylthiazol-2-yl)-2,5-diphenyltetrazolium bromide) assay. For that, *L*. *amazonensis* promastigotes were grown in M199 medium (Hanks salts, 2% human urine, 10% fetal calf serum, gentamicin), at 26 ºC, until stationary phase (approximately 3 days). The protozoa were transferred to 96 well culture plates (1 x 10^6^/ well). Sextuplicates of a serial dilution of free polB, PBCAnp-polB and PBCAnp were added to the plates, followed by a 24h incubation period (26°C)., To evaluate the resulting cell viability, 25 μL of MTT (5 mg/mL) added in each well preceded another incubation at 37 ºC for 4 hours. The formed crystals were solubilized with isopropanol and analyzed on a spectrophotometer at 570 nm. Positive and negative growth controls were obtained with promastigotes without treatment and treated with amphotericin B 25 μg/mL, respectively. The survival percentage was calculated by the Eq (3), based on the optical density (OD) obtained. The IC50 was calculated by the Eq (4).

%Survival=[(ODsample–ODnegctrl)/(ODposctrl–ODnegctrl)]x100 (3)

Where: “neg ctrl” is negative control, and “pos ctrl” is positive control.

%Survival=B+(T−B)/(1+10^((LogIC50–Log[PolB])*HillSlope)) (4)

Where: “T” (Top) and “B” (Bottom) are plateaus in the units of the Y axis (%Survival), “[PolB]” is concentration of polB, and “HillSlope” describes the steepness of the family of curves.

#### Cell viability of macrophages

This assay was a modification of the method described in ISO (*International Organization for Standardization*) 10993–5 for fibroblasts [19]. J774A.1 macrophages were resuspended in complete RPMI 1640 medium (10% inactivated fetal bovine serum, 100 UI/ penicillin, and 100 mg/mL streptomycin).

Quintuplicates of 50 μL of serially diluted free polB, PBCAnp-polB, and PBCAnp were added to 96 well culture plates. Then, 4 x 10^5^ cells/ well were transferred, and these plates were incubated for 24 hours. After that, 25 μL of MTT solution (5 mg/mL) was added, and the plate was incubated again (37°C for 4 hours). 100 μL of isopropyl alcohol was added to solubilize the crystals before analysis (spectrophotometer, 570 nm). The culture medium containing macrophages were considered 100% viable and cells treated with DMSO 10% as 0% of viability. The survival percentage was calculated by Eq (3), based on the optical density (OD) obtained. According to the ISO document, a substance is considered cytotoxic when cell viability is lower than 70%

#### Leishmanicidal activity on infected macrophages by *L*. *amazonensis*

The cultivated macrophages (1 x 10^6^ cells, 1 mL) were transferred to 13 mm coverslips that were inside culture plate wells (24 wells). After incubation (24 hours/ 37°C/ 5% CO_2_), macrophages were infected with promastigotes of *L*. *amazonensis* (5 protozoa/ macrophage cell) and incubated again (4 hours/ 32°C/ 5% CO_2_). The culture was washed to remove non-internalized promastigotes, followed by formulation sample addition and new incubation (24 hours/ 32°C/ 5% CO_2_). After that, cells were gently washed with RPMI 1640 medium, fixed on the coverslips with methanol and stained with a rapid panoptic dye. The infected cells, non-infected cells, and number of amastigotes per cells were counted by microscopic examination of stained coverslips (300 cells per coverslip). This assay was realized in triplicate for each sample. Controls: negative control of infection = untreated macrophage cells; positive control of infection = macrophage cells treated with promastigotes and no samples; positive control of leishmanicidal effect = macrophages treated with promastigotes, followed by amphotericin B treatment.

#### Minimum inhibitory concentration (MIC)

This assay was realized by microdilution method as described by Clinical and Laboratory Standards Institute (CLSI) [20], using *Pseudomonas aeruginosa* ATCC 27853, *Escherichia coli* ATCC 25922, and *Klebsiella pneumoniae* clinical isolate (donated by Clinical Hospital of UNICAMP). The inoculum was prepared by making a saline suspension of the colonies selected from the agar plate of 18–24 hours culture. The turbidity of the suspension was adjusted using the 0.5 McFarland turbidity standard (BaSO_4_) corresponding to approximately 1 to 2 x 10^8^ *Escherichia coli* ATCC 25922 [21]. In a 96-well plate, serial dilution of polB, PBCAnp-polB, and PBCAnp was performed, maintaining the controls: 100% growth (bacteria only), 100% of death (bacteria killed with ethanol) and sterility (Müller-Hinton broth only). The plate was incubated for 24 hours—37°C. After that period, the plate was read visually, and then the optical density measured on the RChisto Infinite M200 PRO plate reader at 625 nm. Positive and negative growth controls were obtained with bacteria without treatment and treated with polB 16 μg/mL, respectively. The survival percentage was calculated by the Eq (3), based on the optical density (OD) obtained.

#### Statistical methods

Calibration curves were determined by linear regression analysis, accepted only if R-squared ≥ 0.99. The difference between two independent means was determined by Student's t-test, with a significance level of 0.05 for a two-tailed test. To compare more than two means we used analysis of variance (ANOVA).

## Results and discussion

Control PBCAnp were formulated by emulsion polymerization method (PBCAnp), as described in methods. Nanoparticle yield was 83.2 + 2.1%, based on dry weight. For the drug-loaded formulation, 5 mg/mL of polB was adsorbed on the newly formed PBCAnp by non-covalent attachment to the dextran outer layer (PBCAnp-polB). Both formulations were characterized and tested *in vitro* to evaluate their antimicrobial and cytotoxicity potential.

### PBCAnp characterization

The formulations showed reproducible size (Table 1), around 200 nm, regardless of polB addition (t = 0 days, t-test, p = 0.51). Size distribution presented a polydispersity index (PDI) of less than 0.2, adequate for pharmaceutical formulations. NPs maintained their size during the stability study, performed up to 45 days, with no significant statistical difference between intragroup means (Anova test, PBCAnp p = 0.11; PBCAnp-polB p = 0.34). According to literature, this size range is adequate to enhance macrophage phagocytic activity and activation of the complement system [22]. The D10 value is higher than 100 nm, which means that 10% of total particles are under the reported this particle size value. In our case, this is a desirable D10, since particles smaller than 100 nm may have modified physical and chemical properties, leading to a greater potential for toxicity [23,24].

**Table 1:** Physicochemical characterization of nanoparticles.

	PBCAnp			PBCAnp-polB		
	Day 1	Day 15	Day 45	Day 1	Day 15	Day 45
**Size (nm)**	216,9 ± 19,2	211,2 ± 20,7	201,2 ± 13,4	216,5 ± 25,9	215,2 ± 25,4	202,9 ± 15,7
**PDI**	0,167 ± 0,054	0,179 ± 0,046	0,156 ± 0,042	0,172 ± 0,050	0,183 ± 0,053	0,152 ± 0,029
**D10 (nm)**	133,3 ± 10,5	129,4 ± 8,7	127,3 ± 5,2	131,9 ± 11,4	126,8 ± 6,7	129,5 ± 8,3
**D50 (nm)**	232,3 ± 24,8	222,1 ± 24,4	210,3 ± 12,7	235,9 ± 33,5	231,1 ± 31,8	216,0 ± 17,9
**D90 (nm)**	416,2 ± 84,8	418,2 ± 90,3	372,7 ± 58,7	440,7 ± 105,3	448,8 ± 113,9	369,8 ± 45,8
**ZP (mV)**	-18,11 ± 3,35	-15,85 ± 1,78	-2,18 ± 0,46	1,79 ± 0,17	1,99 ± 0,13	1,80 ± 0,19

Control poly butyl-cyanoacrylate nanoparticles (PBCAnp) and nanoparticles loaded with polymyxin B 5 mg/mL (PBCAnp-polB) hydrodynamic diameter measurements (1–45 days, 4–8°C, standard deviation correspond to triplicate of formulations analyzed by triplicate measurements), represented by: Z average (size); particle size distribution D10, D50, and D90 correspond to 10%, 50%, and 90% of total particles under the reported particle size value, respectively; polydispersity index (PDI). The former data and Zeta potential (ZP) were obtained with Dynamic Light Scattering (DLS) equipment.

**Fig 1:**
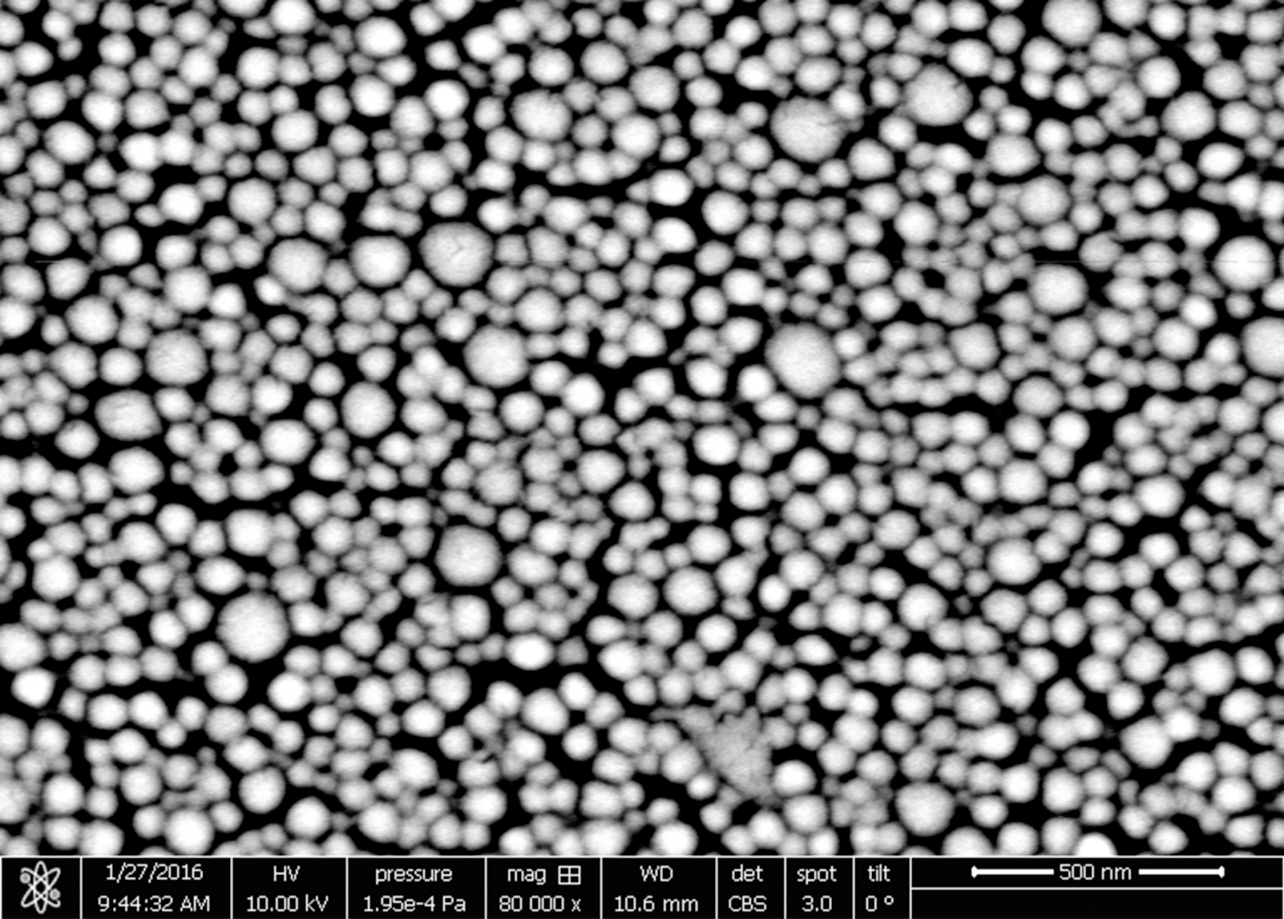
Scanning electron micrograph picture of control poly butyl-cyanoacrylate nanoparticles (PBCAnp). Samples were dehydrated on a silica wafer at room temperature, coated with gold-palladium and analyzed by scanning electronic microscope JEOL JSM-6340-F with magnification x 80,000, confirming the spherical morphology.

Morphological analysis showed that the PBCAnp has a spherical shape (Fig 1), validating the use of DLS measurements that are based in the spherical model for calculations. The spherical shape observed by SEM is in agreement with that found by Bootz and colleagues, as well as the decrease in particle size when compared to that measured by DLS [25]. The size decrease happens due to the removal of water (drying) in sample preparation.

The medium Zeta potential goes from negative (-18.11 ± 5.97 PBCAnp) to positive values (1.79 ± 0.17 PBCAnp-polB) (Table 1) indicating adsorption of the cationic drug on the anionic surface of the NPs and the presence of its free form in solution. The potential shift was expected in polB NPs, and the resulted Zeta potential is not considered enough for pure electric colloidal stability [26]. However, steric stabilization with dextran is sufficient to conserve physical stability [27], confirmed with size maintenance for up to 45 days (Table 1).

NTA size measurements were similar among formulations (Table 2; t-test, p = 0.18), with D10 values above 100 nm, as observed with DLS technique. The mean particle size and D values were higher when measured by DLS (around 200 nm) than those by NTA (around 170 nm), as expected. DLS measures size by volume, whereas larger particles largely contribute to the scattering plot. NTA traces individual particles, and a few larger particles has little effect on the precision of the size measure [28]. Despite the significant statistical difference (t-test, p = 0.66), the values of particles/mL are in the same order of magnitude. NTA does not offer high precision in this concentration measurement [28].

**Table 2:** Particle size and concentration of nanoparticles by Nanoparticle Tracking Analysis (NTA).

Formulation	PBCAnp	PBCAnp-polB
**Particles/ mL**	2.00 (± 0.00) x 10^12^	1.60 (± 0.10) x 10^12^
**Size (nm)**	169.0 ± 1.1	166.0 ± 3.7
**D10 (nm)**	114.0 ± 1.3	112.0 ± 3.1
**D50 (nm)**	147.0 ± 1.9	150.0 ± 5.1
**D90 (nm)**	225.0 ± 5.2	204.0 ± 3.8

Nanoparticles diluted in ultrapure water on 1: 1000 proportion, triplicate measurements, day 1. Control poly butyl-cyanoacrylate nanoparticles (PBCAnp) and nanoparticles loaded with polymyxin B 5 mg/mL (PBCAnp-polB); particle size distribution D10, D50, and D90 correspond to 10%, 50%, and 90% of total particles under the reported particle size value, respectively.

PolB was incorporated in NPs by electrostatic adsorption. In this case, the more drug in the external medium the higher the adsorption [29]. In accordance, previous tests with 1 mg/mL of drug presented a low adsorption to the NPs (216 μg/mL, IE = 21,6%), but 5mg/mL resulted in approximately four times the amount of bounded drug, with a small decrease in incorporation efficiency index (885 μg/mL, IE = 17.7%). However, as the concentration gets too high, adsorption gets close to saturation and encapsulation rate drops more, such as we observed with 10 mg/mL of polB (1050 μg/mL, IE = 10.5%). Therefore, our studies continued with 5 mg/mL of polB added to nanoparticles (named PBCAnp-polB). The efficiency of incorporation was stable: PBCAnp-polB presented 17.7% (885 μg/mL) on t = 1 day, and 16.8% (840 μg/mL) on t = 45 days (t-test, p = 0.99). PBCAnp-polB resulted in approximately 2.4 x 10^5^ molecules of polB per nanoparticle. The experimental drug loading for PBCAnp-polB was 10.53% as compared to a theoretical value of 8.76%, due to unbound polymer (removed in the filtration step).

**Fig 2:**
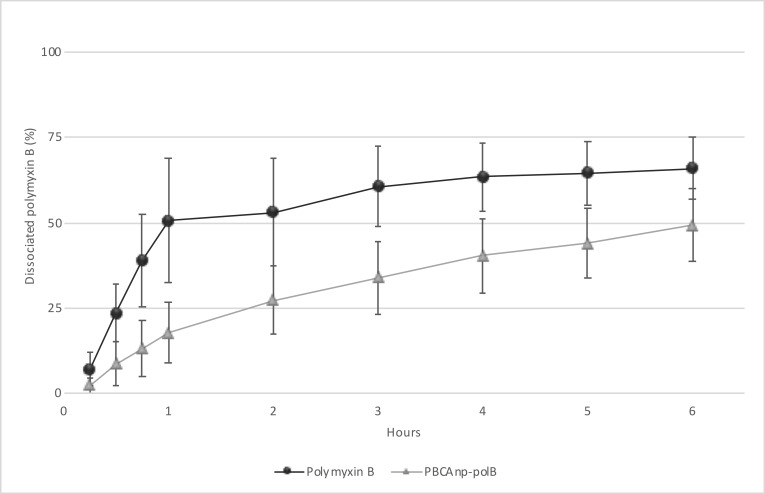
Cumulative release profile of free polymyxin B and poly butyl-cyanoacrylate nanoparticles with polymyxin B 5 mg/mL (PBCAnp-polB). Analysis performed in vertical diffusion cells (6 hours, 37°C, PBS buffer, pH 7.4, n = 6). X axis = hours, Y axis = percentage of dissociated polymyxin B.

We evaluated drug release profile of PBCAnp-polB in PBS buffer pH 7.4 at 37° C (Fig 2). Noteworthy, free polB had no immediate release profile. The cellulose membrane used to separate the test compartments is widely used with proteins and described as having low binding with them, in addition to having 12–14 kDa membrane pores, which are at least 12 times larger than polB (1,023 kDa). Therefore, delayed time can be attributed to drug diffusion rate through the membrane. 50% of free polB was detected in the acceptor compartment after 1 hour of the experiment, while polB in PBCAnp-polB took 6 hours to reach this value. This release profile confirms a sustained release of polB from NPs, adequate for prolonged antimicrobial effect.

### *In vitro* evaluation of leishmanicidal activity of PBCAnp: Free protozoa

**Fig 3:**
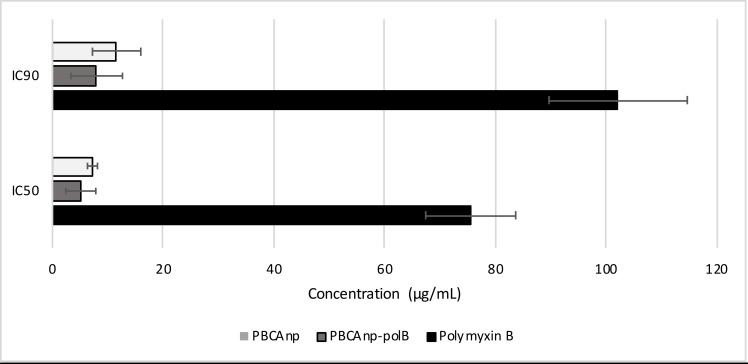
Leishmanicidal IC50 and IC90 concentrations of samples against *L*. *amazonensis*. *L*. *amazonensis* was treated for 24 hours (26°C) with control n-butyl cyanoacrylate nanoparticles (PBCAnp) and nanoparticles loaded with polymyxin B 5 mg/mL (PBCAnp-polB), and analyzed by MTT method: the microtiter plates were evaluated by visible spectroscopy (570 nm). Analysis performed in replicates of six.

Leishmanicidal activity tests were performed *in vitro* with *Leishmania amazonensis* promastigotes. The IC50 value found for free polB was 77.1 μg/mL (59.2 μM) (Fig 3). As a reference, the standard drug meglumine antimoniate presented an average IC50 value of 49 to 60 μg/mL, which was measured in reference strains of the same species. It is important to note that the current model of *in vitro* susceptibility testing may undermine leishmanicidal efficacy, since these peptides can be inactivated by fetal bovine serum and high saline concentration in culture media [30–32]. Unfortunately, high concentration of bovine serum is essential for *Leishmania amazonensis* adequate growth. Previous attempts from our group to diminish serum concentration did not reach satisfactory growth density for the used strain. PolB binding to serum also favours its cell entrance without its membrane activity; consequently, degradation can occur and aminoacids serve as nutritional molecules, which may explain the increased growth and high standard deviation of the less concentrated samples. As the quantity increases, free polB probably predominates and inhibits growth (S5 Fig). In addition, several drugs had lower activity in extracellular *Leishmania* forms: amphotericin B had half potency in promastigotes when compared to amastigotes, whereas sodium stibogluconate presented 3 times less potency in the same comparison [33].

Fig 3 shows that PBCAnp-polB formulation reached IC50 of 6.0 μg/mL (4.6 μM, NP dilution = 1: 830), a high IC50 decrease, compared with the free drug. However, PBCAnp also killed protozoa, (dilution of NP at IC50 = 1: 620) and was largely responsible for the leishmanicidal effect of PBCAnp-polB. In accordance with our results, control PBCAnp were described as active against promastigotes of *Leishmania amazonensis* [34], but the related paper did not evaluate coated NPs, like ours. Leishmanicidal activity of NPs tailored with a similar polymer (poly isohexylcyanoacrylate) was also described in infected macrophages with amastigotes from *Leishmania donovani* [35].

### *In vitro* evaluation of leishmanicidal activity of PBCAnp: macrophages infected with protozoa

**Fig 4:**
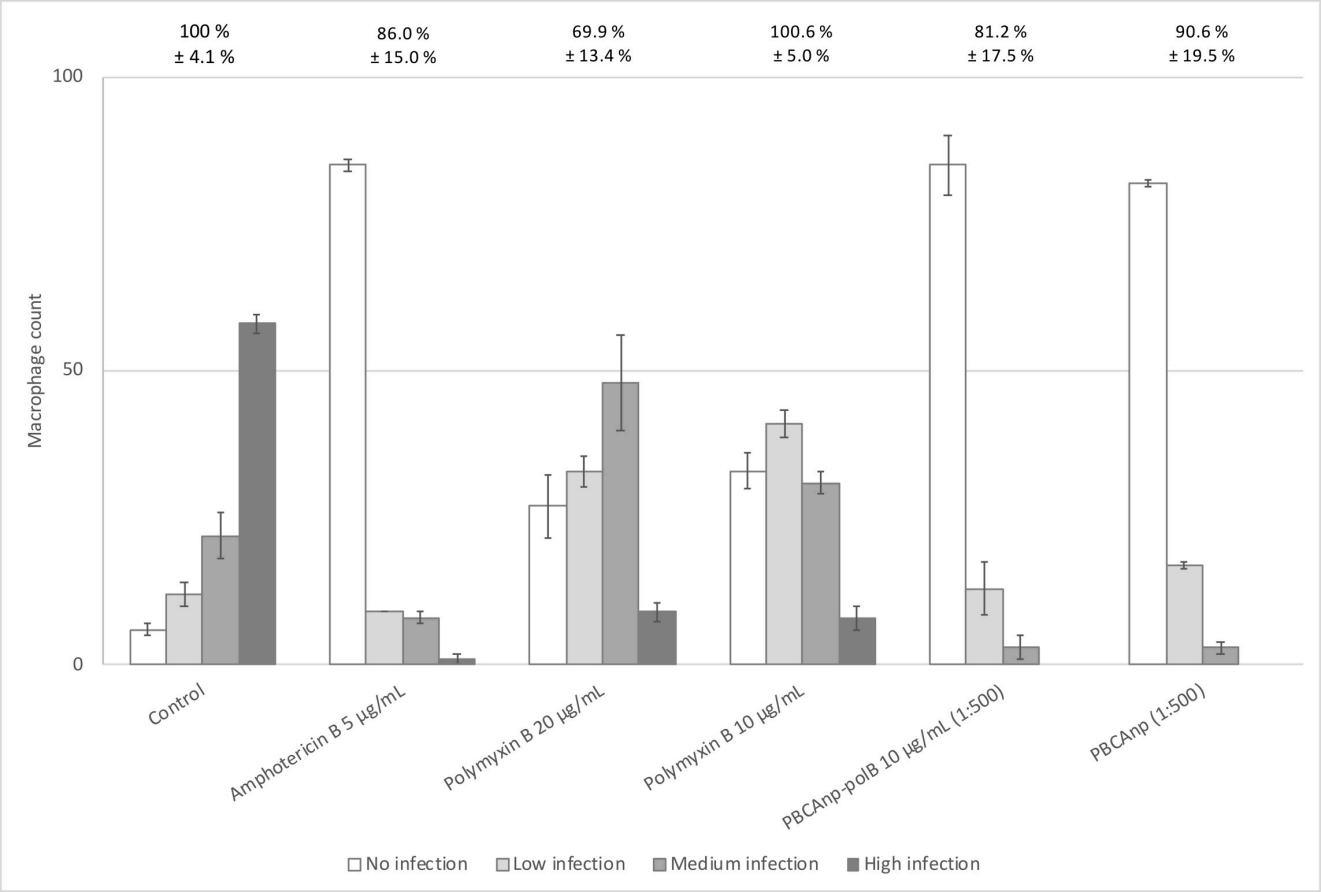
Treatment of J774 macrophages infected with *L*. *amazonensis*. At first, macrophage viability under drug and formulation exposure was determined according to methodology; average results and standard deviation of sextuplicates of each treatment are reported above the 100% line of the graph. Treatments that resulted in cell viability below 70% were considered cytotoxic. Then, treatment of infected macrophages with samples were performed as described in methodology. Controls included infected macrophages without treatment (negative leishmanicidal activity, “control” in the graph) and treatment with amphotericin B (positive leishmanicidal activity). Results from the treatment are represented by bars, which relate to the counting of macrophages, normalized to percentage of viability and separated by infectivity level (legend squares): 1–3 parasites per cell are low infection, 4–6 parasites per cell are medium infection, up to 7 parasites per cell is high infection. Standard deviations of bars correspond to triplicates of each treatment. PBCAnp = control n-butyl cyanoacrylate nanoparticles; PBCAnp-polB nanoparticles loaded with polymyxin B 5 mg/mL.

Firstly, we performed cell viability tests in non-infected J774 macrophages with 10 and 20 μg/mL of polB, free or loaded in NPs, which are equivalent to 2 and 4-fold the IC50 of PBCAnp-polB (Fig 4, values above the 100% line graph). Samples with 10 μg/mL of polB were well tolerated by the cells, but the 20 μg/mL ones presented a 30% decline (free polB) to 81% decline (PBCAnp-polB) in macrophage viability. Although extensively used in macrophage cultures to neutralize lipopolysaccharides, polB lipopolysaccharide-binding concentration is lower than the ones used in this study [36]. The enhanced citotoxicity of the nanostructured drug at the highest concentration derived from the carrier, since control NPs at the respective dilution also presented similar decline (PBCAnp 1:250, 88%). Published papers showed that PBCAnp was not cytotoxic at 15 μg/mL to rat glioma cell lines [37], equivalent to what we found for J744 macrophages (16,8 μg/mL of PBCAnp, equivalent to 1:500 dilution in Fig 4). The same macrophage citotoxicity was found for uninfected murine peritoneal macrophages (50% viability at 16 μg/mL of PBCAnp) in a previous work from our group [35]; other researchers also detected a moderate cytotoxic effect (approximately 60% viability) to THP-1 macrophages at concentrations above 50 μg/mL [9].

Macrophages without treatment (negative leishmanicidal activity, “control”, Fig 4)) presented 58% of highly infected cells (with up to 7 parasites per cell), and only 6% of cells without infection. Amphotericin B, the second choice treatment of leishmaniasis [37], was used as a positive leishmanicidal control and resulted in 85% of uninfected cells, with practically no highly infected macrophages (1%). We found no reports of leishmanicidal profile of amphotericin B regarding macrophage infection levels for comparison.

All treatments decreased highly infected macrophages and increased non-infected cell numbers, compared to the infection control (t test, p < 0.05). Nanoparticle samples (1:500 dilution) reached the average of cleared cells from the one obtained with amphotericin B; the polB adsorption did not changed counting averages. Higher nanoparticle concentrations were toxic to macrophages, therefore were not tested.

PolB treatment with 10 μg/mL had similar behaviour of 20 μg/mL, with no statistical difference, except for low infected counting. Therefore, our results show that increments in polB concentration were not enough to increase its leishmanicidal efficacy.

### *In vitro* evaluation of the antibacterial potential of formulations

As described before, a cutaneous lesion caused by *Leishmania* are frequently contaminated by bacteria. This contamination can lead to a more serious secondary infection, or even worse the leishmaniasis, like *Pseudomonas aeruginosa*. Therefore, the microbicidal activity of the formulations was tested.

The minimum inhibitory concentration used of polB and PBCAnp-polB was 2 μg/mL, in triplicate (*P*. *aeruginosa* ATCC 27853, *E*. *coli* ATCC 25922, and *K*. *pneumoniae* clinical isolate), by visual determination. Since is a two-fold dilution method, the accurate MIC value is bigger than 1, up to 2 μg/mL (1< MIC ≤ 2 μg/mL), as previously reported by other groups [38,39]. All visual determinations corroborated with O.D. analysis (S4 Fig).

Incorporation of polB in NPs did not change MIC results, which is in accordance with the lack of antimicrobial activity from the carrier (at the same dilutions tested for the loaded version). Noteworthy, the incorporation of polB was close to 20%, which still leaves 80% of free drug; the bound polB was released after 6–7 hours in PBS, but it could present different kinetics when applied to microbial culture media. Therefore, this test confirms that polB remains active and bound polB is released fast enough to maintain the 24-hour result from the polB alone.

### Conclusion

The IC50 (against promastigotes) of free polymyxin B (polB) was reported for the first time, such as its intracellular antiparasitic efficacy. The antimicrobial greatly decreased intracellular protozoa number in macrophages, which shows its potential as adjuvant therapy, but it was not capable of a relevant macrophage clearance.

PBCAnp formulations had a low PDI, with a reproducible and stable size, adequate for macrophage targeting. PBCAnp-polB presented a coating of approximately 2.4 x 10^5^ molecules of polB per unit, with a sustained release profile. PolB did not change NP toxicity to protozoa or macrophages. The optimal PBCAnp in pharmaceutics aspects present antileishmanial activity by itself, superposing polB activity. However, as discussed, serum presence in culture media could have underestimated polB efficacy.

PBCAnp did not modify the antibacterial activity of polB at safe dilutions to macrophages, which makes it possible to use them in antibacterial therapeutics for Gram-negative infections or even contaminated ulcers caused by cutaneous and mucosal *Leishmania*. As an example, *K*. *pneumoniae* has been found in lesions caused by leishmaniasis, as well as *P*. *aeruginosa*, which may be related to disease morbidity and require specific treatment [5].

Considering that PBCAnp can also carry drugs inside their cores, this formulation can easily serve as a carrier for leishmanicidal drugs, helping to prevent or treat concomitant bacterial infections.

## Supporting information

S1 Fig Calibration curve of albumin (BSA) and polymyxin B. Curves were performed for polymyxin B dosage calculations in nanoparticles, quantified with the bicinchoninic kit, which is based on colorimetric reaction detected by UV-vis spectroscopy (560 nm). The assay range was 1.9–20 μg/mL. There was a significant difference (t-test, p = 0.57), between curve inclinations, so the polymyxin B curve was used for sample dosage calculations. The method was reproducible (SD less than 0.009), with no interference from the matrix (OD of control NP supernatant had the same reading as blank–water).
(TIF)

S2 Fig Size distribution of poly n-butyl cyanoacrylate nanoparticles (PBCAnp). Dynamic light scattering (DLS) graphs generated during the stability study (0, 15 and 45 days; 4–8°C). (A) Size distribution by intensity. (B) Correlogram of sample size analysis, with adequate correlation coefficients (all above 0.7). Formulations were performed in triplicate, with each time point analysed in triplicate.
(TIF)

S3 Fig Size distribution of poly n-butyl cyanoacrylate nanoparticles with polymyxin B 5 mg/mL (PBCAnp-p5). Dynamic light scattering (DLS) graphs generated during the stability study (0, 15 and 45 days; 4–8°C). (A) Size distribution by intensity. (B) Correlogram of sample size analysis, with adequate correlation coefficients (all above 0.7). Formulations were performed in triplicate, with each time point analysed in triplicate.
(TIF)

S4 Fig **Survival curves of (A) *K*. *pneumoniae*, (B) *P aeruginosa* and (C) *E*. *coli*.** Bacteria were treated for 24 hours (37°C) with polymyxin B, control n-butyl cyanoacrylate nanoparticles (PBCAnp) and nanoparticles loaded with polymyxin B 5 mg/mL (PBCAnp-polB). The microtiter plates were evaluated by visible spectroscopy (625 nm). The analyses were performed in triplicate.
(TIF)

S5 Fig Survival curves of *L. amazonensis* promastigotes. *L*. *amazonensis* was treated for 24 hours (26°C) with (A) polymyxin B, (B) control n-butyl cyanoacrylate nanoparticles (PBCAnp) and nanoparticles loaded with polymyxin B 5 mg/mL (PBCAnp-polB). The microtiter plates were evaluated by visible spectroscopy after MTT assay to calculate viability percentages (570 nm). Wells with parasites only were considered as 100% growth. Analysis performed in replicates of six. * p = 0.001.
(TIF)
