# Supplementary figures and images for: Biodegradable nanocarriers coated with polymyxin B: Evaluation of leishmanicidal and antibacterial potential
Source: PLoS Negl Trop Dis. 2019 May 1;13(5):e0007388. doi: 10.1371/journal.pntd.0007388 (PMC6513107; doi:10.1371/journal.pntd.0007388)

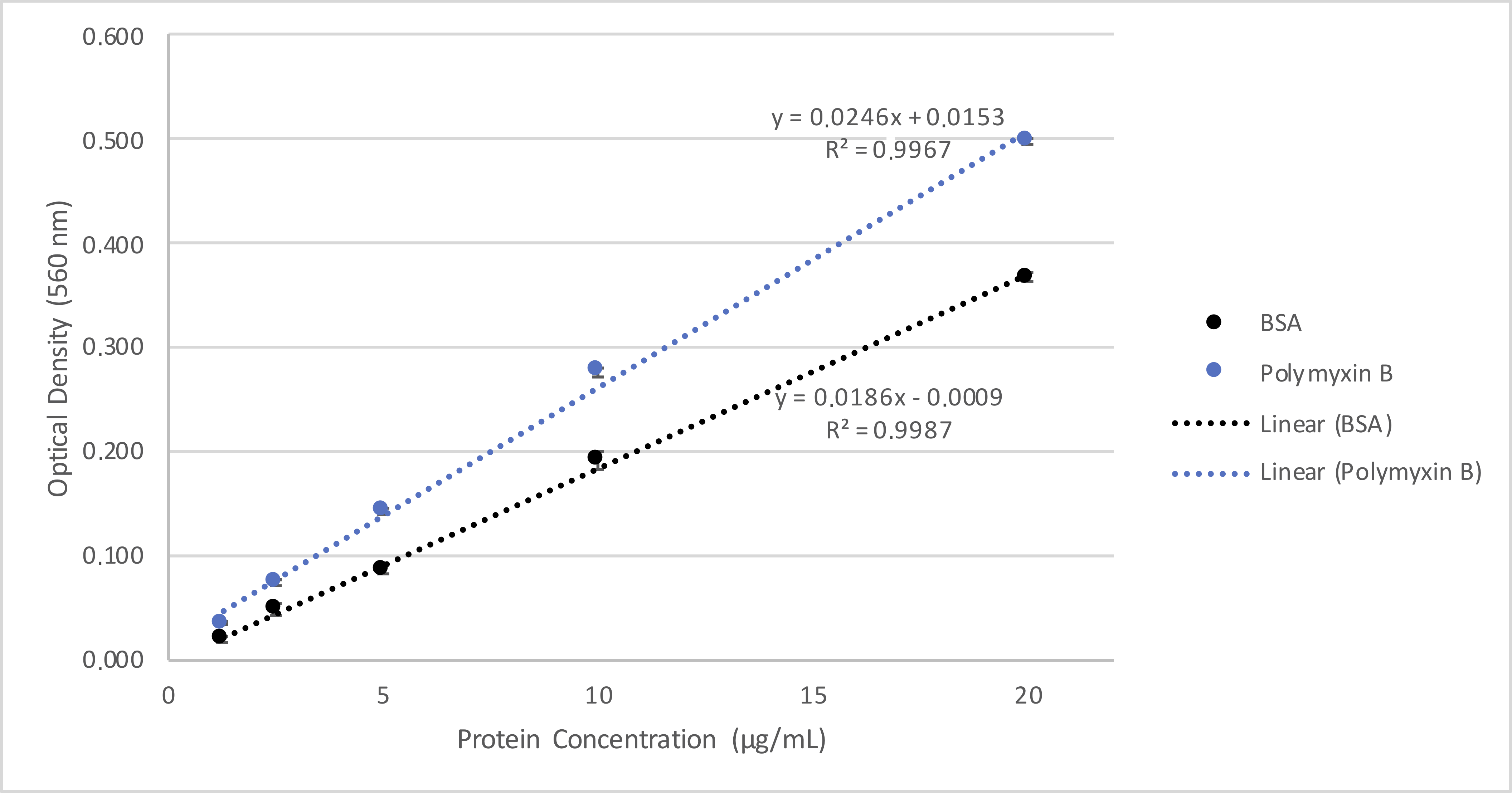

Supplement: S1 Fig — Curves were performed for polymyxin B dosage calculations in nanoparticles, quantified with the bicinchoninic kit, which is based on colorimetric reaction detected by UV-vis spectroscopy (560 nm). The assay range was 1.9–20 μg/mL. There was a significant difference (t-test, p = 0.57), between curve inclinations, so the polymyxin B curve was used for sample dosage calculations. The method was reproducible (SD less than 0.009), with no interference from the matrix (OD of control NP supernatant had the same reading as blank–water). (TIF) [file pntd.0007388.s001.tif]

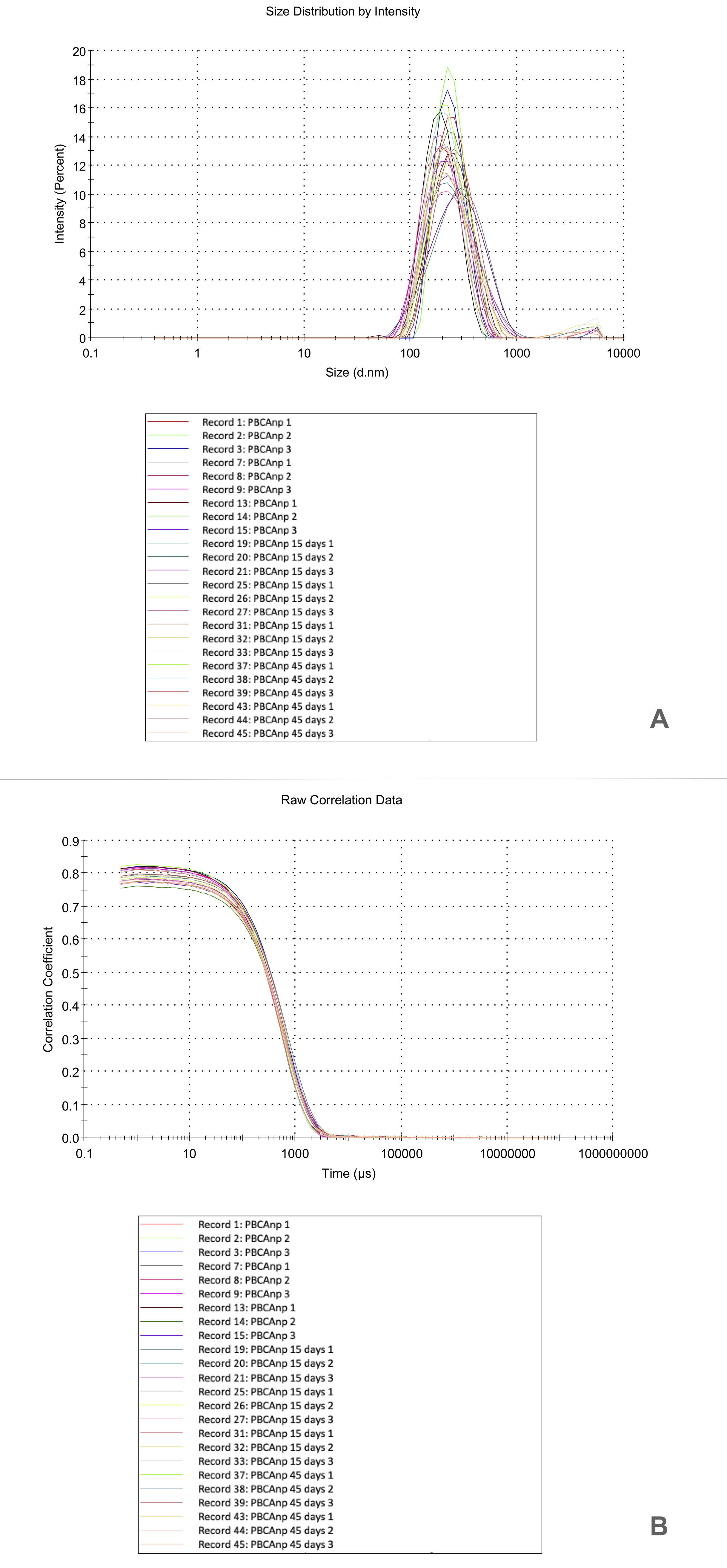

Supplement: S2 Fig — Dynamic light scattering (DLS) graphs generated during the stability study (0, 15 and 45 days; 4–8°C). (A) Size distribution by intensity. (B) Correlogram of sample size analysis, with adequate correlation coefficients (all above 0.7). Formulations were performed in triplicate, with each time point analysed in triplicate. (TIF) [file pntd.0007388.s002.tif]

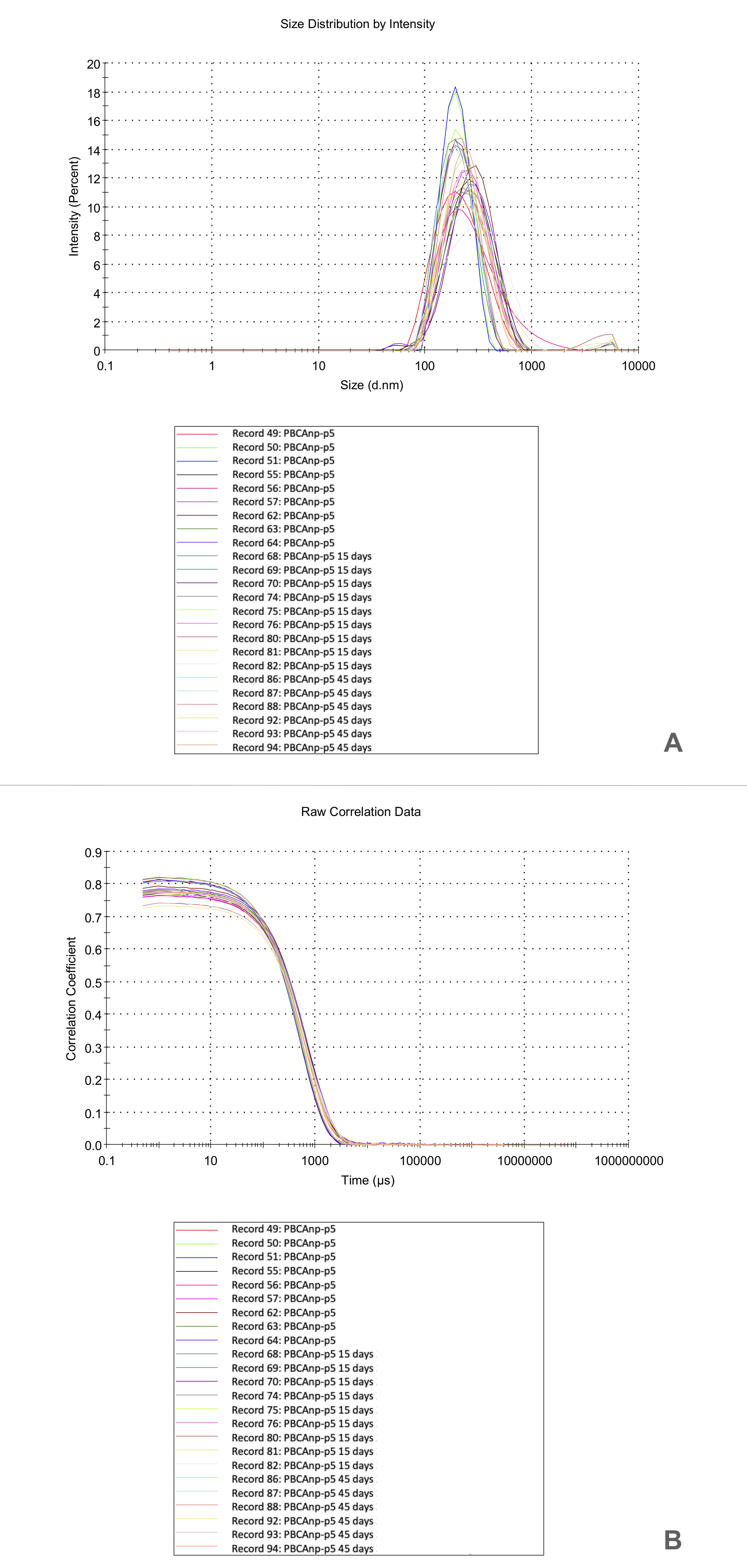

Supplement: S3 Fig — Dynamic light scattering (DLS) graphs generated during the stability study (0, 15 and 45 days; 4–8°C). (A) Size distribution by intensity. (B) Correlogram of sample size analysis, with adequate correlation coefficients (all above 0.7). Formulations were performed in triplicate, with each time point analysed in triplicate. (TIF) [file pntd.0007388.s003.tif]

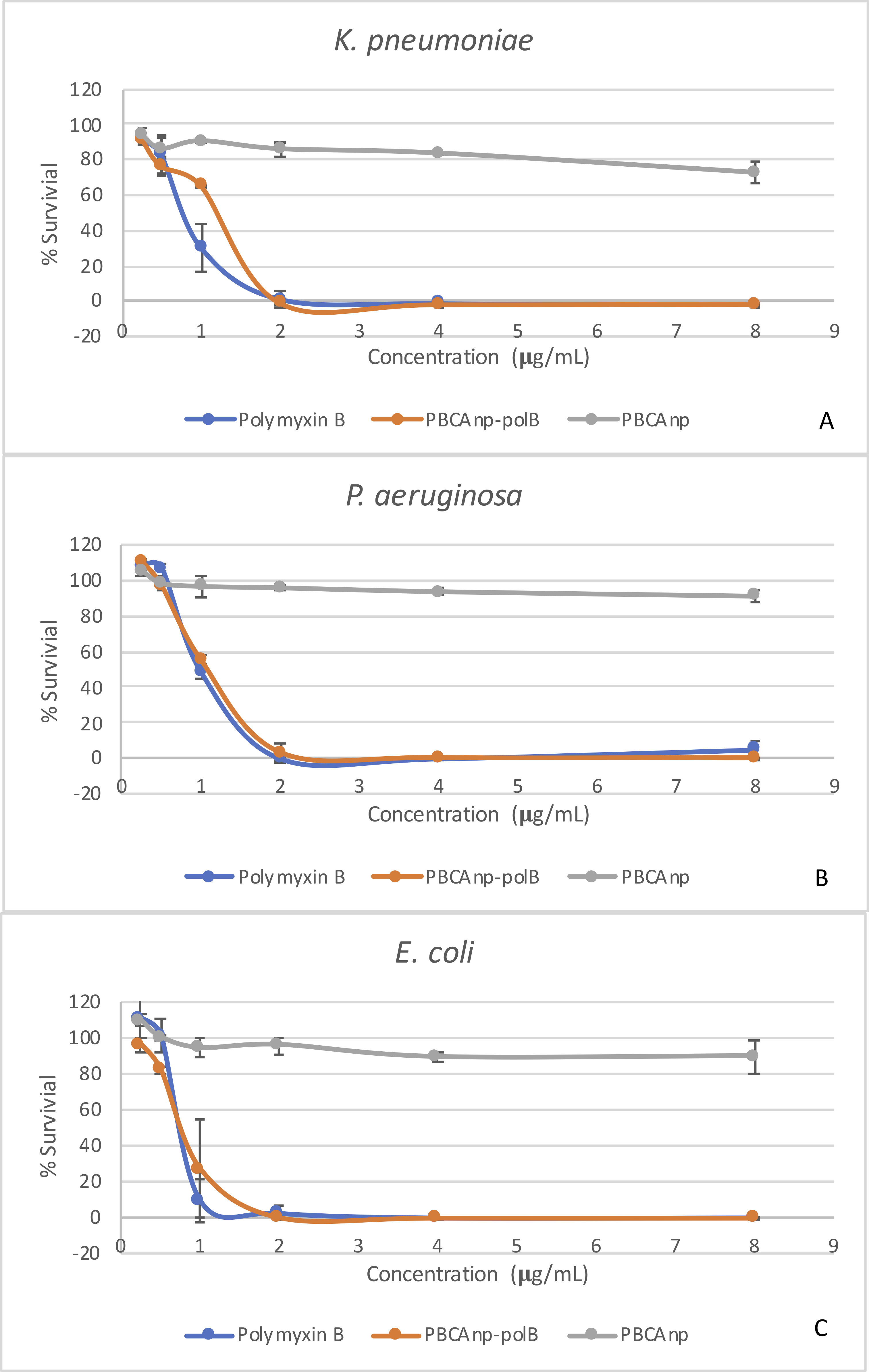

Supplement: S4 Fig — Survival curves of (A) K. pneumoniae, (B) P aeruginosa and (C) E. coli. Bacteria were treated for 24 hours (37°C) with polymyxin B, control n-butyl cyanoacrylate nanoparticles (PBCAnp) and nanoparticles loaded with polymyxin B 5 mg/mL (PBCAnp-polB). The microtiter plates were evaluated by visible spectroscopy (625 nm). The analyses were performed in triplicate. (TIF) [file pntd.0007388.s004.tif]

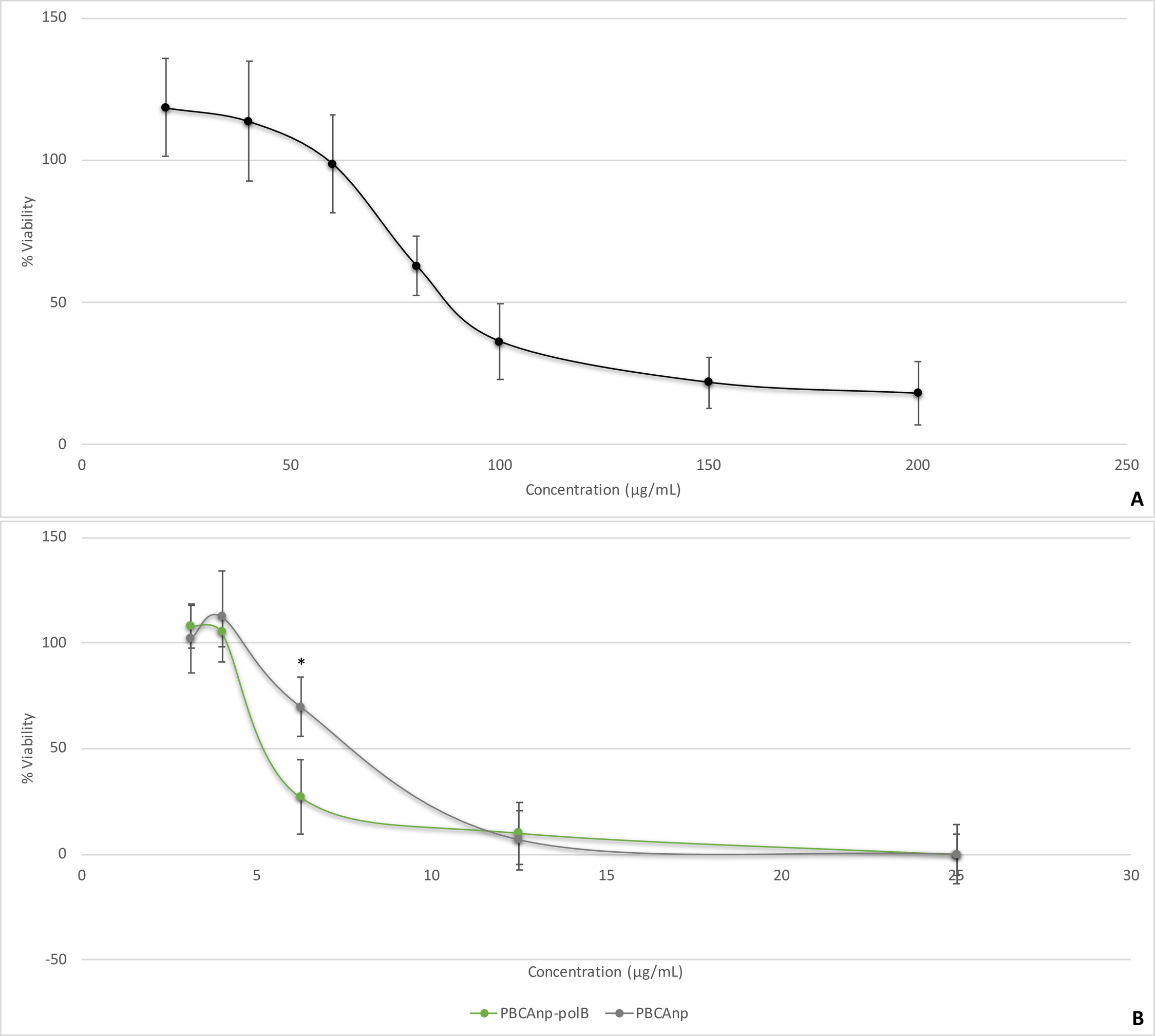

Supplement: S5 Fig — L. amazonensis was treated for 24 hours (26°C) with (A) polymyxin B, (B) control n-butyl cyanoacrylate nanoparticles (PBCAnp) and nanoparticles loaded with polymyxin B 5 mg/mL (PBCAnp-polB). The microtiter plates were evaluated by visible spectroscopy after MTT assay to calculate viability percentages (570 nm). Wells with parasites only were considered as 100% growth. Analysis performed in replicates of six. * p = 0.001. (TIF) [file pntd.0007388.s005.tif]
